# Androgen receptor polyglutamine repeat number: models of selection and disease susceptibility

**DOI:** 10.1111/j.1752-4571.2012.00275.x

**Published:** 2012-06-11

**Authors:** Calen P Ryan, Bernard J Crespi

**Affiliations:** Department of Biological Sciences, Simon Fraser UniversityBurnaby, BC, Canada

**Keywords:** androgens, balancing selection, cancer, disease susceptibility, infertility, sexual conflict, tandem-repeat accumulation

## Abstract

Variation in polyglutamine repeat number in the androgen receptor (AR CAGn) is negatively correlated with the transcription of androgen-responsive genes and is associated with susceptibility to an extensive list of human disease. Only a small portion of the heritability for many of these diseases is explained by conventional SNP-based genome-wide association studies, and the forces shaping AR CAGn among humans remains largely unexplored. Here, we propose evolutionary models for understanding selection at the AR CAG locus, namely balancing selection, sexual conflict, accumulation-selection, and antagonistic pleiotropy. We evaluate these models by examining AR CAGn-linked susceptibility to eight extensively studied diseases representing the diverse physiological roles of androgens, and consider the costs of these diseases by their frequency and fitness effects. Five diseases could contribute to the distribution of AR CAGn observed among contemporary human populations. With support for disease susceptibilities associated with long and short AR CAGn, balancing selection provides a useful model for studying selection at this locus. Gender-specific differences AR CAGn health effects also support this locus as a candidate for sexual conflict over repeat number. Accompanied by the accumulation of AR CAGn in humans, these models help explain the distribution of repeat number in contemporary human populations.

## Introduction

An applied understanding of the evolutionary forces shaping human health and disease susceptibility has profound medical implications, providing clinical insights and suggesting novel, testable hypotheses (Di Rienzo 2006; Nesse 2011). Robust integration of evolutionary theory with human medicine must continue to address topics whose resolution eludes research in each field independently. In particular, identifying the extent and regions of the human genome most directly subjects to balancing selection (Andrés et al. 2009), and mutation-selection balance (Keller and Miller 2006; Haerty and Golding 2010b), and explaining the ‘missing heritability’ of complex diseases (Manolio et al. 2009), have remained problematic, despite their central importance for the interfaces of disease biology with human evolution.

Tandem-repeat polymorphisms can be key components of human phenotypic and disease-related variation (Ashley and Warren 1995; Sutherland and Richards 1995; Koshy and Zoghbi 1997; Reddy and Housman 1997; Gatchel and Zoghbi 2005). These genomic elements are largely undetectable using SNP-based genome-wide association (GWA) studies (Hannan 2010), yet they constitute about 3% of the human genome, inhabit as many as 10–20% of human genes and promotors, exhibit mutation rates that are orders of magnitude higher than those of SNPs, show high levels of within-population variation, and exert graded as well as discrete functional effects on gene expression and activity via a suite of documented molecular mechanisms (Kashi and King 2006; Gemayel et al. 2010; Kelkar et al. 2011). Over 40 human diseases, as well as continuous variation in phenotypes related to morphology, physiology and behavior, have been associated with tandem-repeat variation (Fondon and Garner 2004; Pearson et al. 2005; Fondon et al. 2008; Gemayel et al. 2010). As such, tandem-repeat loci represent excellent candidates for the effects of balancing selection (Mularoni et al. 2010) and mutation-selection balance (Haerty and Golding 2010b), as well as mediating notable potential for rapid, adaptive evolution (Birge et al. 2010).

Two well-studied human tandem-repeat polymorphisms are situated within the X-linked androgen receptor (AR) gene, which codes for a transcription factor that mediates binding of the androgens testosterone (T) and dihydrotestosterone. Androgens play an integral role in the organizational and ontogenic processes involved in sexual differentiation and male-sexual development during embryogenesis (Swerdloff et al. 1992; Chang et al. 2002), although the AR remains widely expressed in a range of tissues in both male and female adults (Ruizeveld de Winter et al. 1991). Among other functions, AR-mediated gene transcription is integral to skeletal (Kenny and Raisz [Bibr b106]), muscular (Dillon et al. 2010), and nerve cell (Arnold and Breedlove 1985; Hammond et al. 2001) development and maintenance, and in the regulation of cognition and behavior (Cherrier and Craft [Bibr b27]; Eisenegger et al. 2011).

Within exon 1 of the AR gene are two trinucleotide repeats (CAG and GGN) whose numbers vary both within and between human populations (Figure S1; Edwards et al. 1992; Ackerman et al. 2012). Independent studies support an inverse relationship between the transcriptional activity of the AR and polyglutamine repeat length (AR CAGn; Kazemi-Esfarjani et al. 1995; Choong et al. 1996; Buchanan et al. 2004), and at least 64 different diseases and phenotypes have been investigated in relation to AR CAGn (Table S1). Given the functionally diverse roles of the AR in human development, physiology, and behavior (Chang et al. 1995; Chang 2002), coupled with more general effects of polyglutamine repeat number on mutability and function (Kashi and King 2006), the spectrum of putative effects of polymorphism in AR CAGn is well founded. However, the factors responsible for shaping the frequency and distribution of AR CAGn, which varies in number between eight and thirty-seven repeats in healthy humans (Zitzmann et al. 2003; Lindstrom et al. 2010), and its potential role in unexplained heritability in those diseases have received little attention (but see Hannan 2010). Here, we suggest AR CAGn as a promising, paradigmatic candidate helping explain missing heritability in human disease mediated by androgen levels, and we propose and evaluate four potential models of selection acting on the AR CAG locus to maintain intermediate repeat frequency in human populations: balancing selection, sexual conflict, accumulation-selection, and antagonistic pleiotropy.

First, balancing selection contributes to human illness when deleterious alleles are maintained in the population through heterozygote advantage or when disease risk results from extremes of bidirectional variation in gene expression (review in Crespi 2010). To the extent that the diseases described here are related to variations in CAGn and the transcriptional activity in the AR, balancing selection should modulate the distribution of AR CAGn within and among populations. Under these conditions, selection will be strongest at the genetic and phenotypic extremes of AR CAGn, and the accompanying fitness costs and benefits should vary with the environment in which the AR is expressed (i.e. genetically, physiologically, ecologically).

Second, sexual conflict may be a selective force acting on AR CAGn, specifically if fitness optima for AR responsivity and polyglutamine repeat number differ between men and women owing to differences in the benefits or disease-related costs of repeat number. A model for sexual selection, antagonistic pleiotropy, and sexual conflict at the AR CAG locus has been proposed (Summers and Crespi 2008), whereby an early advantage to male fertility and reproduction arising from shorter repeat number entails increased risk of cancer for both sexes. While in men the fertility benefits of short repeat number may compensate for the costs of greater prostate cancer risk, the absence of such benefits in women may lead to genomic conflict over repeat number between the sexes (Summers and Crespi 2008). Still, to our knowledge, sex differential fitness costs and the potential for sexual conflict accompanying the remaining 60+ diseases and phenotypes putatively linked to AR CAGn (Table S1) have not yet been proposed. The extent of sexual conflict and the magnitude of the costs arising from conflict over AR CAGn will, like balancing selection, be dictated by the contribution of repeat number to disease susceptibility, etiology, and their impacts on survival and reproduction for each sex.

Third, the expansion of tandem repeats is a common feature in the human genomic landscape, and an important force shaping the frequency of AR CAGn alleles in human populations (Rubinsztein et al. 1995; Gatchel and Zoghbi 2005). Although the mechanisms of accumulation of tandem repeats are becoming clear, the reasons behind the tendency toward expansion and repeat fidelity in humans (compared to other mammalian and primate lineages) are not well understood (Vowles and Amos 2006; Kelkar et al. 2008). Tandem-repeat expansions in protein coding regions are remarkably widespread, even though expansion is frequently associated with disease-related health effects (Buschiazzo and Gemmell 2006). If disease-associated selection is biased against phenotypes arising from longer than average AR CAG repeat number, then in a modified mutation-selection model, selection would primarily oppose the pressures of expansion of repeat number. Repeat number variation may then represent a fluctuating equilibrium between the strength of selection against longer AR CAGn and the propensity toward repeat accumulation, the rate of which is influenced by a collection of factors and which includes, intriguingly, the number and sequence purity of repeats already present at that locus (e.g. Fig. 2 in Buschiazzo and Gemmell 2006).

Finally, the nature and magnitude of phenotypic effects and disease susceptibilities putatively associated with AR CAGn should vary within an individual's lifetime, suggesting the potential for antagonistic pleiotropic effects in any or all of the above models of selection. Traits linked to AR CAGn which favor mating success and fertility early in life may be in conflict with disease-associated costs later on (e.g. Summers and Crespi 2008; Carter and Nguyen 2011); these costs be may appear exaggerated in contemporary societies owing to increases in modal life span (Gurven and Kaplan 2007).

To evaluate these four non-exclusive hypotheses for explaining the distribution and variability in AR CAGn within and among human populations, we review eight of the best-studied diseases from a range of phenotypic classes putatively associated with AR repeat length, evaluate the evidence for and against associations between repeat length and disease, and appraise the relative strength and direction of selection for each disease. We also consider the health costs accompanying longer or shorter extremes in repeat number, the potential for sexual conflict arising from sex differences in the costs of, and the possible effects of antagonistic pleiotropy on, AR CAGn-associated disease. We conclude by discussing the accumulation of AR CAG repeat number in the human lineage in the context of human evolution and human disease susceptibility, and the potential role of AR CAGn as a component of the missing heritability of diseases linked to circulating androgen levels.

## Methods

### Literature search

We obtained data on the role of AR CAGn in human health and disease, using three online databases and one comprehensive review (Rajender et al. 2007) to compile a list of phenotypes and diseases with published, putative associations with AR CAG repeat length (Table S1). Database sources were the following: the AR Mutations Database (Gottlieb et al. 2004; ARDB; http://androgendb.mcgill.ca/, accessed 28 May, 2011), the online mendelian inheritance in man (OMIM) database (MIM ID *313700, accessed 30 May, 2011), and the genetic association database (GAD; http://geneticassociationdb.nih.gov, accessed 21 May, 2011). All phenotypes associated with polymorphisms in AR CAGn were initially included, but mutations or polymorphisms at other loci in the AR (e.g. AR GGNn) were discarded. Using phenotypes and references obtained from these databases, subsequent searches focused on disease phenotypes and risk associations, and studies investigating the molecular structure and function of the AR were included only for interpreting mechanisms of pathology or their role in selection on AR CAGn frequency in human populations. Additional literature was collected using Web of Science, Google Scholar, and PubMed databases using combinations of the search terms: ‘AR’ or ‘AR’, ‘CAG’, ‘CAG repeat’ or ‘Polyglutamine repeat’, and individual disease names (e.g. ‘Prostate Cancer’).

### Disease and study selection

To explore how susceptibility to disease risks associated with AR CAGn may shape the frequency of this polymorphism in humans, eight diseases were evaluated as follows: four putatively linked to longer repeat length, four to shorter repeat length. Necessary disease inclusion criteria were that the disease be referred to in at least three of the four main sources (the three databases and review) mentioned above and that it be the subject of at least one large-scale (500+ participants) study or meta–analysis examining its relationship to AR CAGn. Diseases that matched these criteria and were the focus of ≥10 independent studies were considered, as was colorectal cancer, for which we deemed the relationship between this disease and AR CAGn to be well supported by two particularly large (>3500 subjects for colon cancer; >1800 subjects for rectal cancer) case–control studies (Slattery et al. 2005). We were left with 12 remaining disease susceptibilities that we considered of equal interest but beyond the scope of the current study; therefore, we included eight diseases, which well represent the diversity of androgen function and disease class phenotypes described in Table S1. Of the remaining diseases not included but of interest are endometrial cancer, ovarian cancer, polycystic ovarian syndrome, and muscle mass/obesity and type II diabetes/metabolic syndrome. We consider the eight diseases ultimately chosen to evaluate hypotheses about the models of selection on AR CAGn illustrative and characteristic of disease relationships with the AR, and potential focal points for further research on the role of AR CAGn in missing heritability and on the models of selection proposed.

### Strength of selection

Although fitness costs of disease under ancestral conditions may be only loosely associated with clinical severity in contemporary human populations (Di Rienzo 2006), we evaluated the capacity for a disease to act as a selective agent based on three criteria, all of which would be reasonably expected to affect lifetime reproductive success. These were as follows: the frequency of disease occurrence; the average age of onset of the disease; and the effect of the disease on survival, fertility, and reproduction. Common diseases with profound reproductive or survivability effects, and with earlier onset in contemporary human populations, were predicted to have the greatest capacity to exert direct selective effects on AR CAGn. However, negative selection against susceptibility alleles associated with typically ‘late-onset’ diseases is likely greater than previously believed, as a result of marked variability in age at onset and a number of factors other than direct selection (e.g. effects on survival and reproduction of kin; Pavard and Metcalf 2007). Additionally, extensive allelic variability and the potential for rapid and reversible changes in tandem-repeat purity and number (Kashi et al. 1997) make exaggerated selection in short timescales a distinct possibility at the AR CAGn locus. While the prevalence of a number of the diseases discussed (e.g. osteoporosis, cardiac diseases, some types of cancer) in past populations has been difficult to estimate, data emerging from paleoepidemiological studies support the presence of these diseases in human history, although contributing environmental factors in those populations may have differed (Zimmerman 1993; Mays 1998; Faltas 2010). Factors with the capacity to modulate the severity or progression of a disease, such as diet or lifestyle, may play an important role in disease-related costs and are described further in Table 1 and Table S1.

**Table 1:** Susceptibility for eight diseases putatively associated with AR exon 1 polyglutamine repeat number (AR CAGn)

Disease putatively associated with AR CAG	Disease prevalence	Age of onset	Susceptible sex	Health effects	Risk factors	References
Longer						
Spinal bulbar muscular atrophy	Rare	Mid–late reproductive	Males	Survival and reproduction	Androgens levels, pneumonia	1–8
Infertility	Common	Early reproductive	Males	Reproduction	Ethnicity, SHBG, epigenetics	9–20
Breast cancer	Very common	Late reproductive	Predominantly females	Survival	Other genes, hormone therapy, family history, parity	21–39
Osteoporosis, decrease BMD	Very common	Late reproductive	Both sexes	Survival	Age, gender, SHBG	40–45
Shorter						
Prostate cancer	Very common	Mid-Late reproductive	Males	Survival and reproduction	Androgen levels, other genes	46–73
Cardiac diseases	Very common	Mid reproductive	Both sexes	Survival	Diet, lifestyle	74–80
Colorectal cancer	Common	Mid-late reproductive	Both sexes*	Survival	Diet, gender, other genes/hormones	81–84
Cognition and behaviour disorders	Common	Pre-reproductive	Males	Survival?	Age, gender, environment	85–93

AR, androgen receptor; BMD, bone mass density; SHBG, sex hormone-binding globulin.

Diseases grouped by proposed direction of association, and prevalence is based on data from contemporary American society (≤0.0001/100 people = rare, 0.01–0.1/100 = common, and ≥0.1/100 = very common. Age of onset of disease pertains to the age at which health effects most likely become evident relative to reproductive age. Effects of disease on health and the sex most susceptible are described, and possible risk factors are provided based on the references provided.

* Direction of association with colorectal cancer may differ for each sex, see text.

References: 1. Amato et al. (1993). 2. Kazemi-Esfarjani et al. (1995). 3. Mariotti et al. (2000). 4. Dejager et al. (2002). 5. Greenland et al. (2004). 6. Atsuta et al. (2006). 7. Katsuno et al. (2010). 8. La Spada et al. (1991). 9. Asatiani et al. (2003). 10. Tut et al. (1997). 11. Thangaraj et al. (2002). 12. Dowsing et al. (1999). 13. Davis-Dao et al. (2007). 14. Komori et al. (1999). 15. Lim et al. (2000). 16. von Eckardstein et al. (2001). 17. Meyts et al. (2002). 18. Yong et al. (2003). 19. Lazaros et al. (2008). 20. Verhoeven et al. (2010). 21. Comings et al. (2003). 22. Díaz-Chico et al. (2007). 23. Elhaji et al. (2001). 24. Ferro et al. (2002). 25. Giguère et al. (2001). 26. Goode et al. (2002). 27. Haiman et al. (2002). 28. Haiman et al. (2003). 29. Hao et al. (2010). 30. Iobagiu et al. (2005). 31. Kaaks et al. (2005). 32. Lillie et al. (2004). 33. Maclean et al. (2004). 34. López-Otín and Diamandis (1998). 35. Rebbeck et al. (1999). 36. Spurdle et al. (2005). 37. Suter et al. (2003). 38. Wang et al. (2005). 39. Yu et al. (2000). 40. Gennari et al. (2007). 41. Guadalupe-Grau et al. (2010). 42. Langdahl et al. (2003). 43. Limer et al. (2009). 44. Tofteng et al. (2003). 45. Zitzmann et al. (2001a,b). 46. Coetzee and Ross (1994). 47. Monroe et al. (1995). 48. Tilley et al. (1996). 49. Giovannucci et al. (1997). 50. Stanford et al. (1997). 51. Pettaway (1999). 52. Beilin et al. (2001). 53. Kittles et al. (2001). 54. Latil et al. (2001). 55. Panz et al. (2001). 56. Shibata et al. (2001). 57. Azzouzi et al. (2002). 58. Chang et al. (2002). 59. Mononen et al. (2002). 60. Schatzl et al. (2002). 61. dos Santos et al. (2003). 62. Buchanan et al. (2004). 63. Gilligan et al. (2004). 64. Giwercman et al. (2004). 65. Zeegers et al. (2004). 66. Alvarado et al. (2005). 67. Forrest et al. (2005). 68. Freedman et al. (2005). 69. Platz et al. (2005). 70. Andersson et al. (2006). 71. Summers and Crespi (2008). 72. Lindstrom et al. (2010). 73. Kumar et al. (2011). 74. Hersberger et al. (2005). 75. Pausova et al. (2010). 76. Zitzmann et al. (2001a). 77. Alevizaki et al. (2003). 78. Lind et al. (2008). 79. Page et al. (2006). 80. Rexrode et al. (2008). 81. Slattery et al. (2005). 82. Gillessen et al. (2010). 83. Ferro et al. (2000). 84. Di Fabio et al. (2009). 85. Cheng et al. (2006). 86. Rajender et al. (2008). 87. Jönsson et al. (2001). 88. Westberg et al. (2009). 89. Manuck et al. (2010). 90. Fondon et al. (2008). 91. Yaffe et al. (2003). 92. Seidman et al. (2001). 93. Colangelo et al. (2007).

## Results

### Disease risk and longer AR CAGn

#### Spinal bulbar muscular atrophy

Spinal bulbar muscular atrophy (SBMA) shows an unequivocal relationship with AR CAGn. Patients with SBMA invariably have longer repeat number than is observed in the general population, typically between 38 and 62 repeats (La Spada et al. 1991; Amato et al. 1993; Brooks and Fischbeck 1995). Symptoms include late-onset muscular weakness and atrophy, frequently accompanied by androgen insensitivity and hypogonadism (Dejager et al. 2002; Palazzolo et al. 2008), believed to be a result of AR protein aggregation resulting in apoptosis of affected cells (Grierson et al. 1999; Ellerby et al. 2002; Vismara et al. 2009). Women may act as carriers of higher repeat number, experiencing mild if any symptoms of the disease, and toxicity appears to remain low even among women homozygous for high numbers of repeats (Mariotti et al. 2000; Greenland et al. 2004; Katsuno et al. 2010). Disease onset is typically later in life (30–60 years of age), although longer AR CAGn is predictive of earlier disease onset, which is often preceded by less severe symptoms including muscle fatigue and cramping (Atsuta et al. 2006). Despite the relatively rare nature of this disease (roughly 1/40 000 men), repeat numbers in the SBMA range bear formidable negative health effects. Risk of aspiration pneumonia (the most common cause of death in SBMA patients; Katsuno et al. 2010), muscle degeneration, and loss of mobility would likely have been strongly selected against under most ancestral conditions. The rescuing effect of a second less toxic allele with shorter repeat number in women, accompanied by the reduction in symptoms in homozygotes, means that the costs of high repeat number associated with this disease differ for men and women, conditions which would contribute to sexual conflict over AR CAG repeat length. There are no known fitness benefits associated with repeat numbers in the SBMA range.

#### Male infertility

The essential role of androgens in male virility and spermatogenesis (Collins and Chang 2002), and the association between SBMA and infertility (e.g. Arbizu et al. 1983) have led to a number of investigations into the differences in AR sensitivity arising from variations in CAGn and idiopathic male infertility (e.g. Table 1 and references therein). Repeat number at the AR CAGn among infertile patients has been variously found to be longer (e.g. Tut et al. 1997; Dowsing et al. 1999; Lim et al. 2000; von Eckardstein et al. 2001; Davis-Dao et al. 2007; Nenonen et al. 2010), shorter (Komori et al. 1999; Nenonen et al. 2010), or not significantly different (e.g. Dadze et al. 2000; Meyts et al. 2002; Thangaraj et al. 2002; Yong et al. 2003) from those of controls, with ethnic or population level differences potentially confounding the results. A large-scale meta-analysis provides good support for a link between longer AR CAGn and infertility (Davis-Dao et al. 2007), but the average contribution of each additional repeat to infertility has not been empirically demonstrated. Still, the actual difference between patients and controls is likely to underestimate the effect of repeat number, given the fact that an unknown proportion of patients with repeat numbers in the shorter, ‘normal’ range will be infertile because of other unknown causes (Davis-Dao et al. 2007). A non-linear relationship between infertility and AR CAGn has also been proposed, such that men with longer *or shorter* AR CAGn than the median (22–23 repeats) are at a 20% increased risk of infertility (Nenonen et al. 2010). If this pattern is true, then stabilizing selection around intermediate repeat frequency could arise from male infertility alone. Given the relative commonness of male infertility (estimated to be approximately 7%; Meacham et al. 2007), and the age-independent and thus potentially profound effects of AR CAGn on male lifetime reproductive success and fitness, longer (and possibly shorter) AR CAGn should be under strong selection from infertility in men.

#### Breast cancer

Endogenous androgen steroid levels have been recognized as modulating factors associated with breast cancer (BC) (Adams 1998; Ferro et al. 2002; Kaaks et al. 2005), and between 60% and 70% of BCs express the AR as well as androgen-dependent proteins (e.g. PSA and GCDFP-15; Díaz-Chico et al. 2007). *In vitro* studies support a protective effect of androgens on hormone-independent BC-cell lineage proliferation (Di Monaco et al. 1995; Gatto et al. 1996; Szelei et al. 1997), and low premenopausal androgen levels have been associated with susceptibility to this disease (Adams 1998; Wang et al. 2000). Longer AR CAGn has been correlated with BC risk (Giguère et al. 2001; Haiman et al. 2002; Liede et al. 2003; Suter et al. 2003; but see Hao et al. 2010), younger age at onset (Rebbeck et al. 1999) and tumor aggressiveness (Yu et al. 2000) and grade (Elhaji et al. 2001; Maclean et al. 2004). Because the effects of AR CAGn on BC risk interact with a number of other factors, including nutrition (Kaaks et al. 2005), hormone treatment (Suter et al. 2003; Lillie et al. 2004), polymorphisms at other loci (Rebbeck et al. 1999; Suter et al. 2003), family history (Rebbeck et al. 1999; Haiman et al. 2002), and ethnicity (with Caucasians showing the highest risk and the longest average repeat number; Altekruse et al. 1975), it is difficult to infer the magnitude of selection against longer AR CAGn resulting from BC. Still, the high prevalence (lifetime risk approximately 12%; Altekruse et al. 1975) and variance in age at onset of the disease (Pavard and Metcalf 2007), and the importance of alloparental care from post-reproductive women suggest that the fitness costs of susceptibility to BC in ancestral environments could have been significant and could have contributed to the distribution in AR CAGn repeat number we see in contemporary populations. Although a positive relationship between repeat number and the occurrence and grade of BC has also been observed in men (Maclean et al. 2004), selection owing to disease susceptibility in this sex is unlikely to contribute to AR CAGn owing to the very low occurrence of this disease in men (approximately 0.13%; Altekruse et al. 1975).

#### Osteoporosis and bone mass density

The general role of androgens in bone metabolism, loss of bone mass in cases of hypogonadism, and reduction in bone turnover with testosterone treatment all lead to predictions for a decrease in bone mass density (BMD) and increase in osteoporosis (femoral neck BMD <0.56 g/cm^2^) with longer AR CAGn (Zitzmann et al. 2001b; Zitzmann 2009). A relationship between polyglutamine repeat lengths among premenopausal (but not postmenopausal) women with lower BMD has been shown (Yamada et al. 2004), as has a relationship between BMD and AR CAGn on the longer of the two alleles in women, with significantly longer AR CAGn among female patients compared to controls (Langdahl et al. 2003). In healthy men, AR CAGn is a negative predictor of BMD, and the effect of age on bone loss is greater in subjects with longer repeat length (22–31 repeats) compared to those with shorter repeat lengths (14–21 repeats; Zitzmann et al. 2001b). Several studies have reported the opposite, however, finding both a negative (Limer et al. 2009) or both positive and negative relationship between BMD, bone mineral content (BMC), and AR CAGn, but only in conjunction with AR GGNn (another amino acid repeat polymorphisms in the AR; Guadalupe-Grau et al. 2010), or under the modulation of steroid hormone binding globulin (SHBG; Tofteng et al. 2003).

Independent of other factors, longer AR CAGn may contribute to BMD and risk of osteoporosis, and osteoporosis prevalence differs considerably between ethnic groups as well as between the sexes (Melton 2001). The Third National Health and Nutrition Examination Study recorded 20% of postmenopausal Caucasian women as osteoporotic, compared to only 5% in African American women. By contrast, only 4% of Caucasian men over 50 years of age were defined as osteoporotic, compared to 2% of African American men (Looker et al. 1997). These differences in prevalence mirror ethnic AR CAGn frequencies, with African-Americans possessing shorter repeat lengths (Figure S1), which supports a role for longer AR CAGn in osteoporosis risk.

Although often considered to be a ‘disease of civilization’ (Karasik 2008), a body of archeological evidence suggests that osteoporosis may be more common among human history than once believed (Mays 1998 and references therein; Poulsen et al. 2001; Cho and Stout 2011; but see Agarwal and Grynpas 1996). In fact, poor nutrition and extended lactation may have contributed to even higher rates of osteoporosis or earlier onset of age-related losses in BMD in some regions, despite the positive effect of greater physical activity in those populations (Turner-Walker et al. 2001). While hip fractures in past populations appear to be rare (possibly owing to the shorter overall lifespan or selection against low BMD earlier on in life; Mays 1998), other bone-density related fractures might have been more common and accompanied by poorer functional outcomes (Mays 2006). Also, the relatively porous nature of human vertebral bone (compared to our closest living relatives) makes it particularly susceptible to fracture with even modest losses in BMD (Cotter et al. 2011), and the health consequences of these types of acute trauma and debilitation no doubt exceeded those of current mechanized societies. The poor functional outcomes accompanying even relatively ‘minor’ fractures associated with losses in BMD, potentially earlier age at onset from deficient childhood nutrition or extended lactation, and the importance of alloparental care from post-reproductive women (Hawkes 2003) suggest that osteoporosis associated with longer AR CAGn has the capacity to act as a selective force AR CAG repeat number frequency. As for a modulating effect of AR CAGn in the missing heritability of osteoporosis (Karasik 2011), at least one intriguing study has identified heritable components of canine skeletal morphology with glutamine repeat number at another locus: heritability not detected using traditional SNP-based approaches (Fondon and Garner 2004), pointing to the importance of tandem repeats in bone structure and function.

## Disease risk and shorter AR CAGn

### Prostate cancer

Evidence strongly supports a role for androgens and AR CAGn in prostate cancer (PC) risk and progression. Castrated or hypogonadic men (including men suffering SBMA) rarely develop prostate cancer, and chronic exogenous androgen administration in rats can induce the disease (Henderson and Feigelson 2000; Hsing et al. 2008). Prostate cancer progression is sensitive to androgen deprivation (a common therapy), and a crucial stage in disease progression is the evolution of androgen-independent cancer cell lineages (Tilley et al. 1996; Henderson and Feigelson 2000; Grönberg 2003; Ross et al. 2005). Inheritance patterns also support a contribution of X-linked genes (which include the AR), with brothers of individuals succumbing to the disease showing greater risk of developing prostate cancer themselves than sons of individuals with the disease (Monroe et al. 1995). Shorter AR CAGn repeat number has been associated with disease risk (Irvine et al. 1995; Panz et al. 2001; Andersson et al. 2006; but see Forrest et al. 2005), age at onset/diagnosis (Beilin et al. 2001; Latil et al. 2001; dos Santos et al. 2003), and prostate cancer grade, stage, metastasis and fatality resulting from the disease (Giovannucci et al. 1997; Hakimi et al. 1997; Shibata et al. 2001). Shortening of AR CAGn is also commonly associated with PC progression (Alvarado et al. 2005), and the AR itself has become a key target for therapeutic research (Berger et al. 2011). Additionally, ethnic differences in AR CAGn (like BC and osteoporosis) mirror racial susceptibility to prostate cancer, with men of African origin displaying the shortest CAGn and the highest incidence of prostate cancer, with the opposite being true of Asians (Figure S1; Edwards et al. 1992; Coetzee and Ross 1994; Pettaway 1999; Kittles et al. 2001; Panz et al. 2001). A 2004 meta-analysis confirmed a significant difference between cases and controls, although the differences do appear to be modest (<1 repeat difference between patients and controls; Zeegers et al. 2004).

In contrast to the patterns described above, the largest study to examine prostate cancer and AR CAGn (Lindstrom et al. 2010) did not detect any relationship between these two traits, nor did several other large-scale studies multi-ethnic cohort study (Mononen et al. 2002; Freedman et al. 2005). One explanation for the difference between earlier and more recent studies has been diagnostic technologies for identifying prostate cancer in its early stages. The widespread use of prostate-specific antigen (PSA) beginning in the early nineties has shifted detection to less aggressive manifestations and earlier stages of the disease, which do not always progress to advanced stages of the disease, or may do so much more slowly (Platz et al. 2005).

There is no doubt that factors other than AR CAGn are important in prostate cancer risk and disease etiology (e.g. AR GGNn; Hakimi et al. 1997; Stanford et al. 1997). Still, the relatively robust connection between AR CAGn and disease susceptibility and the high occurrence of the disease in men (approximately 16% lifetime risk; Altekruse et al. 1975), coupled with successful reproduction to relatively old ages in human men, mean that even modest increases in the susceptibility to prostate cancer could engender considerable fitness costs and be an important contributor to AR CAGn polymorphism frequencies in contemporary human populations. Antagonistic pleiotropy arising from selection for prostate-expressed genes and their implication on prostate cancer have been formally proposed (Summers and Crespi 2008), but the promising role of AR CAGn in explaining missing heritability by modulating prostate cancer risk and progression via interactions with circulating androgen levels may deserve increased attention.

#### Cardiac diseases and atherosclerosis

Testosterone has been suggested as a contributing factor to the higher rates of atherosclerosis and cardiac disease among men than among women and may affect a range of risk factors contributing to susceptibility to these diseases (Hanke et al. 2001; Weidemann and Hanke 2002; Wu and Eckardstein [Bibr b190]). Higher sympathetic vasomotor tone, blood pressure, and intra-abdominal fat, all factors known to contribute to cardiac disease rate, were found among French Canadian boys (aged 12–18 years) with shorter AR CAGn when compared to boys with longer CAGn (Pausova et al. 2010). Similarly, European men with shorter repeat length associated positively with obesity and stenosis of the arteries (Alevizaki et al. 2003) and negatively with high-density lipoprotein cholesterol levels (Hersberger et al. 2005) and flow-mediated dilatation (Zitzmann et al. 2001a). Shorter AR CAGn also correlated with higher LDL-cholesterol in Spanish women (Rodríguez-González et al. 2009), and ventricular hypertrophy in men (Lind et al. 2008), suggesting an adverse affect of short AR CAGn for cardiac and atherosclerotic diseases. However, Page et al. (2006) failed to detect any relationship between body mass, heart disease, and HDL, even over a 15-year follow-up period, nor did another study on American women (Rexrode et al. 2008). Several protective parameters for cardiac disease, including lower body fat mass and insulin levels, have also been associated with shorter AR CAGn (Zitzmann et al. 2003; but see Gustafson et al. 2003), making interpreting the role of AR CAGn in cardiac diseases difficult. With all European studies supporting an effect of AR CAGn on at least some cardiac disease risk factors (Zitzmann et al. 2001a, 2003; Alevizaki et al. 2003; Hersberger et al. 2005), and one small and one large American study finding no effect (Page et al. 2006; Rexrode et al. 2008), potential population-level differences merit further consideration. Although extremely common in contemporary populations (nearly one-half and one-third lifetime disease risk by the age of 40 for American men and women, respectively), heart disease and atherosclerosis are largely modulated by diet and lifestyle, which also explain some of the missing heritability and population level differences. The mismatch between current and ancestral diet and lifestyle is a major contributor to cardiac and vascular diseases, making selection from these disease susceptibilities unlikely to contribute significantly to AR CAGn frequencies in ancestral human populations. Still, cardiac diseases and atherosclerosis appear common among certain ancient Chinese and Egyptian social classes as well as among some ethnic groups (e.g. the Inuit) and have been identified in 5000+-year-old mummified remains (Murphy et al. 2003), suggesting that these diseases should not be dismissed outright as ‘diseases of civilization’ (David et al. 2010; Allam et al. 2011).

#### Colon and rectal cancer

Androgens regulate growth and differentiation in colon and rectal tissue, and there is support for an association between low testosterone levels and colon cancer in laboratory animals (Xiao et al. 2007; Gu et al. 2009). Studies in animals suggest a protective role of androgens in colon tumorigenesis (Ferro et al. 2002), and prostate cancer patients undergoing long-term androgen deprivation therapy were at a greater risk of developing colorectal cancer (Gillessen et al. 2010). While longer AR CAGn corresponds to the risk of colon cancer in men, longer repeat length appears to be protective in women (Slattery et al. 2005). Women with long repeat number in another polymorphic gene, the β-estrogen receptor, in addition to long AR CAGn, also had a higher risk of disease than women with shorter repeat numbers for both alleles (Slattery et al. 2005). It is worth noting that African American men show lower lifetime risk for the disease compared to Caucasian women and that this relationship is reversed for African Amerian and Caucasian women (Altekruse et al. 1975), corresponding to shorter and longer AR CAGn, respectively. These findings suggest that susceptibility to colon cancer is associated with both longer *and* shorter AR CAGn, *depending on gender*, which correspond to ethnic level differences in mean AR CAG repeat number (Figure S1). The protective effects of shorter AR CAGn in men versus the increased risk of disease in women means that optimal repeat number for AR CAGn with respect to colon cancer alone may differ for each sex. The relatively high frequency of colon cancer occurrence in contemporary populations (approximately 5% lifetime risk in Americans; Altekruse et al. 1975) implies that, in addition to being a strong candidate disease for sexual conflict over optimal AR CAGn, susceptibility to colorectal cancer could contribute to variation in AR CAGn among human populations. Colorectal cancer is another case where the sources of heritable susceptibility remains unclear (Lascorz et al. 2010), and where modulation of the effects of androgens on AR CAGn may be an important factor in susceptibility to this disease (Slattery 2006).

#### Cognitive and behavioral disorders

Although not mutually exclusive, at least three psychological traits show some support for a role of the AR CAGn: (i) aggression, violence, and criminal activity; (ii) cognitive functioning and general intelligence; and (iii) depression. Other socio-behavioral traits, including social and sexual behavior, have been linked to AR CAGn as well as to other repeat polymorphisms in non-human mammals (Hammock and Young 2005; Fondon et al. 2008).

Consistent, though, non-significant trends toward aggressive and dominant behavior were first associated with shorter AR CAGn (Jönsson et al. 2001) and subsequently spurred interest in this area. AR CAGn has since been significantly correlated with both aggression and risky behaviors among boys (Vermeersch et al. 2010) and inmates (Aluja et al. 2011) with shorter repeat length, and a study on Taiwanese criminals found that a significantly larger proportion of violent criminals carried short alleles (<17) than did controls (Cheng et al. 2006). Among Indian men, Rajender et al. (2008) observed significantly shorter repeat length among murderers and rapists than controls, and convicts of both murder and rape had significantly shorter repeat length than criminals who committed murder or rape, but not both. More recently, a study looking at amygdala reactivity among Caucasian American men using fMRI found higher reactivity to facial displays of negative affect among men with shorter AR CAGn (Manuck et al. 2010), although a questionnaire-based study of college students did not detect personality differences with respect to polymorphisms at the AR (Hurd et al. 2011). AR CAGn has also been linked to dominance and status, variables associated with intrasexual competition for mates (Simmons and Roney 2011), and the response of men to potential mates (Roney et al. 2010).

Cognitive functioning in elderly, community-dwelling men was inversely correlated with AR CAGn for all three of the cognitive tests originally examined by Yaffe et al. (2003), but no such relationship has been observed in middle-aged and aging European men (Lee et al. 2010) nor in a sample of healthy Chinese volunteers of varying ages (Kovacs et al. 2009). Manning (2007) has suggested a hypothesis for an affect of AR CAGn on neuronal transmission rate and general intelligence (*g*), which proposes that *g* increases with repeat length observed along the mammalian lineage is constrained in humans by the negative effects of SBMA and impaired sperm production. This ‘gain in function’ for general intelligence with longer AR CAGn has little empirical support, but given the highly expressed nature of the AR in areas of the brain associated with visual and verbal memory (Cherrier and Craft [Bibr b27]), cognitive functioning (Kovacs et al. 2009), and neurological development and neuroprotection (Hammond et al. 2001; Perrin et al. 2008), this line of thinking should not be dismissed and could provide insights into cognitive and disease susceptibility differences observed between the sexes.

In a recent study of adolescent boys, free testosterone levels have been associated with aggressive and non-aggressive risk-taking behaviors, self-esteem, and inversely correlated to depressive symptoms, but these relationships were highly dependent on AR CAGn (Vermeersch et al. 2010). A significant interaction between total testosterone and depressive symptoms has also been observed in men with the short, but not men with long, AR CAGn (Seidman et al. 2001), with similar findings in black (who tend to have shorter AR CAGn lengths), but not white, American men (Colangelo et al. 2007). Collectively, these data suggest that short AR CAGn may contribute to the risk of depression, particularly when testosterone levels are low.

The data described above suggest that men with shorter AR CAGn are more generally intelligent, violent, and aggressive, and less inclined toward depression, but that this relationship may be largely dependant on circulating testosterone levels. The modulating effect of AR CAGn is particularly intriguing, given the reciprocal relationship between dominance and testosterone; testosterone levels not only affect, but are also affected by, dominant social behavior (Mazur and Booth 1998). As a result, the psychological responses to competitive or goal-directed behavior may be mediated by testosterone, but the psychological costs and benefits of high or low testosterone levels may be greater for men with short AR CAGn. Based on these data, it is also interesting to consider a role of sexual selection for cognitive and behavioral traits of testosterone, which may encompass both mood-oriented and cognitive effects modulated by AR CAGn and AR transcriptional sensitivity, although the typically small, multigenic, and environmentally influenced the effect of repeat variation like AR CAGn make it an ongoing challenge to detect their effects (Fondon et al. 2008).

### Disease risk and the accumulation of tandem repeats in the human genome

There is a well-documented increase in coding single tandem repeats, like the AR CAGn, which accompanies the evolution of mammals, primates, and humans (Rubinsztein et al. 1995; Andrés et al. 2004; Vowles and Amos 2006; Kehrer-Sawatzki and Cooper 2007; Mularoni et al. 2010). For the AR CAGn, repeat accumulation along the evolutionary trajectory of mammals is close to exponential (Choong et al. 1998); accumulations that persist in humans, even though long repeat number is frequently associated with pathology, including neurodegenerative disease such as SBMA (Gatchel and Zoghbi 2005). The expansion rate for trinucleotide repeats like the AR CAGn is influenced by sequence repeat purity (Buschiazzo and Gemmell 2006), and while there is evidence for selection acting on trinucleotide repeats (Hancock et al. 2001; Haerty and Golding 2010a), polyglutamine repeats in the human genome are more common and retain a higher degree of sequence fidelity than predicted by neutral expectations alone (Gemayel et al. 2010; Haerty and Golding 2010b; Mularoni et al. 2010). Thus, even though selection should favor point mutations modifying the repeat sequence purity, which would decrease the propensity toward further disease-causing repeat accumulation, repeat sequence in disease-associated coding regions is more highly conserved than in nearby non-coding regions. More intriguing is the fact that tandem repeats with conserved sequences tend to be concentrated on certain amino acids and in certain categories of genes (e.g. glutamine repeats and transcription factors, as for the AR CAGn; Hancock et al. 2001; Gemayel et al. 2010) and that repeat sequence purity, and hence the propensity for repeat accumulation, may differ among human populations (Sobczak and Krzyzosiak 2004), consistent with some adaptive, functional role for repeat accumulation in the human genome.

## Discussion

Balancing selection, sexual conflict, and accumulation-selection are empirically supported as forces with the potential to contribute to AR CAGn frequencies among human populations. These conclusions are based on the causal links between AR CAGn and disease susceptibility, the prevalence the diseases in question, their age at onset, their effects on survival, fertility and reproduction, as well as sex-dependent differences in the putative fitness costs associated with each disease. Of the eight disease susceptibilities evaluated, there is empirical support for a role of AR CAGn in disease susceptibility to five of these diseases: SBMA, infertility, and BC for longer AR CAGn, prostate cancer for shorter AR CAGn, and cancer of the colon for both long and short repeat length, depending on the sex of the carrier. The strong association between osteoporosis and AR CAGn is tempered by equivocal paleopathological data regarding prevalence in past populations (Ekenman et al. 1995; Agarwal and Grynpas 1996; Mays 1998; Karasik 2008), making it more difficult to infer the role of this disease on AR CAGn distribution. The diseases associated with later age at onset (i.e. prostate cancer, BC, osteoporosis; Table 1) could still be significant contributors to AR CAGn distributions owing to the generally underappreciated capacity for negative selection at late-onset disease susceptibility alleles. Variability in age at onset, the contribution of children born to women of 39 years and older in pre-industrial societies (e.g. 6–11% of lifetime reproductive success; Pavard and Metcalf 2007), and indirect contributions of late survival to fitness (e.g. grandmothering; Hawkes 2003; but see Kachel et al. 2011) imply that while antagonistic pleiotropy may be a less important factor to explain AR CAGn distributions than originally hypothesized (Summers and Crespi 2008; Carter and Nguyen 2011), the late-onset diseases discussed still have the capacity to exert effects on lifetime reproductive success. To the extent that repeat number in the AR contributes, either directly or indirectly, to susceptibility to these complex diseases, AR CAGn and comparable microsatellite loci are promising candidates for helping us to explain some of the missing heritability of disease risk not currently accounted for in traditional SNP-based GWA studies.

Compelling evidence for disease risk accompanying AR CAGn at both long and short AR CAGn implies that balancing selection is involved in CAGn number distributions in human populations. To address this hypothesis, the sum benefits, costs, and susceptibilities of long AR CAGn diseases must balance the sum benefits, costs and susceptibilities of short AR CAGn diseases, which should be reflected in the mean number of repeats in the population of interest. Balancing selection at the population level would maintain high levels of intermediate repeat number, with fewer individuals possessing high or low repeat number, one possible explanation for the ‘bell-shaped’ distribution for AR CAGn observed (Figure S1). The variance and mean repeat number in a population will then reflect susceptibilities for each disease and their fitness costs, which must therefore be interpreted in the physiological, geographical, and ecological context in which they have, and presumably are, evolving.

There is also support for sexual conflict over AR CAGn locus, given sex differences in susceptibility and disease type (Summers and Crespi 2008). For SBMA, infertility, and susceptibility to colon cancer, men should favor shorter AR CAGn, whereas women may favor shorter (BC) or longer (colon cancer) repeat lengths. In fact, if the AR CAGn affects susceptibility to colon cancer in men and women differently, then this disease alone could contribute to conflict over repeat number between the sexes. In men, the benefits of short repeat number may exceed the costs (i.e. from prostate cancer), and as there appear to be fewer benefits of short repeat number for women, our findings appear to support a hypothesis of sexual conflict over AR CAGn. The costs and benefits of short repeat length may also differ owing to other sex-dependant modulating factors, such as circulating androgen levels, which could reasonably explain part of the ethnic variation in AR CAGn (Figure S1).

The tendency toward polyglutamine repeat accumulation within mammalian, primate, and human lineages, and the well-documented disease risks accompanying longer AR CAGn (Choong et al. 1998; Gatchel and Zoghbi 2005; Kelkar et al. 2008; Mularoni et al. 2010) provide support for a model of accumulation-selection at this locus. The accumulation of repeats, driven by events such as slippage during replication (Buschiazzo and Gemmell 2006), could be offset in the AR by selection against longer repeat number from SBMA, infertility, and colon cancer and BC. This hypothesis provides an alternative, but not necessarily exclusive, explanation to balancing selection and sexual conflict when considering the distribution of repeat numbers among human populations, particularly if the propensity for repeat accumulation does indeed differ between populations (Sobczak and Krzyzosiak 2004). Our understanding of, and interest in, the functional role and evolutionary context of trinucleotide repeats continues to grow (Vismara et al. 2009; Castel et al. 2010; Haerty and Golding 2010a; Luo et al. 2012), and if the suggestion that accumulation of trinucleotide repeats like the AR CAGn is a non-neutral process, contributing to genetic variability for rapidly evolving traits (Birge et al. 2010), then trade-offs between adaptive trait variation and the costs of disease susceptibility may be pivotal in the proposed accumulation-selection model. If an ‘equilibrium’ of costs and benefits in repeat number exists, then AR CAGn distribution among populations becomes particularly interesting in the light of changing selective pressures and medical intervention in locus-associated diseases. With human intervention in some diseases potentially outpacing others, the outcome over many generations may be changes in median population repeat number and shifts in phenotypes and the susceptibility to other diseases associated with AR CAGn. Given the push toward detailed phenotypic data collection for large clinical cohorts and the implementation of novel evolutionary models to track phenotypic and disease-related changes (Stearns et al. 2010), genes like the AR CAGn may be useful targets in tracing effects of human-induced changes and gene-culture co–evolution.

## Conclusions

Models taking into account balancing selection, sexual conflict, antagonistic pleiotropy, and accumulation-selection will be instrumental to our understanding of disease susceptibility associated with repeat number at the AR CAGn and other loci. Testing these hypotheses requires accurate estimates of disease susceptibility and fitness costs (and benefits) of repeat length, or at least quantitative proxies for these metrics (Polanski et al. 1998), as well as studies of molecular-evolutionary forces affecting such loci within and among human populations. One of the great challenges is a quantitative measure of disease susceptibility and costs from AR CAGn, especially considering genetic correlations and interactions, gene-by-environment interactions, and thus myriad contributions to disease risk and clinical outcomes. Mismatch between the ancestral environments in which repeat number evolved and the current environment, and the differences in disease rates and costs that accompany this mismatch add yet another dimension to an already formidable task.

Still, the challenge of testing evolutionary hypotheses is not restricted to hypotheses of human health and disease (Gluckman et al. 2011), and the AR and CAGn provide a excellent system in which to explore these complex and elusive forms of selection in the human genome. The growing number of sequenced human genomes (The 1000 Genomes Project Consortium 2010; http://www.1000genomes.org), the recent construction of a large-scale, annotated database of expressed trinucleotide repeats like the AR CAGn (Luo et al. 2012), and large, long-term, multigenerational studies (Stearns et al. 2010) may open up new possibilities for studying the evolutionary and functional context of accumulations at the AR CAGn, and for resolving some of the disease-associated consequences of their expansion (Haerty and Golding 2010a).

These kinds of advances may also provide powerful insights into the role of the AR CAGn in the missing heritability of complex diseases and phenotypes modulated by androgens. Tandem repeats such as the AR CAGn may be more informative than SNPs at the individual level owing to their functional role and greater standing genetic variation in human populations, but have been largely neglected in GWA studies owing to the statistical power and high-throughput assays required to incorporate them (Ku et al. 2010). Yet finer-scale analyses of the genetic architecture of the human genome, including tandem repeats like the AR CAGn, are becoming an increasingly important goal in the pursuit of missing heritability for complex phenotypes and disease (Eichler et al. 2010).

A more comprehensive picture of the heritability of human disease susceptibility must also account for interactions between genes and between genes and the environment (Eichler et al. 2010; Stearns et al. 2010). As an evolvable, dynamic, yet robust, interface between cellular responses and the physiological and ecological environment, the endocrine system and its receptors are ideally situated to mediate a wide range of disease susceptibilities and health-related effects. Incorporating tandem repeats, particularly those with known functional roles like those found in the AR CAGn, into the current GWA study framework may unveil genetic and environmental interactions confounding current efforts to explain disease risk and etiology (Hannan 2010; Ku et al. 2010). While understanding the mechanistic and functional consequences of polymorphisms in tandem-repeat number are vital, the evolutionary forces upon which that genetic and functional variation is superimposed are inextricable from phenotypic and disease-associated manifestations. Applied as a component of more comprehensive GWA study design or therapeutically in relation to conventional (e.g. androgen supplementation or ablation) or novel (e.g. targeting instable repeats) personalized disease treatments, tandem repeats like the AR CAGn hold great promise for the effective identification and treatment of disease. In each case, the costs and benefits of polymorphisms in tandem-repeat number variation are fundamentally embedded in their evolutionary legacies.

## Supporting Information

**Figure S1.** AR CAGn distribution among human ethnic populations.

**Table S1.** Phenotypes and diseases of androgens and the AR CAGn.
