# Supplementary material for: Androgen receptor polyglutamine repeat number: models of selection and disease susceptibility
Source: Evol Appl. 2012 Jun 11;6(2):180–96. doi: 10.1111/j.1752-4571.2012.00275.x (PMC3586616; doi:10.1111/j.1752-4571.2012.00275.x)
Supplement: Supplementary file 1 [file eva0006-0180-SD1.docx]

**Appendix A: AR CAGn distribution among human ethnic populations**

**
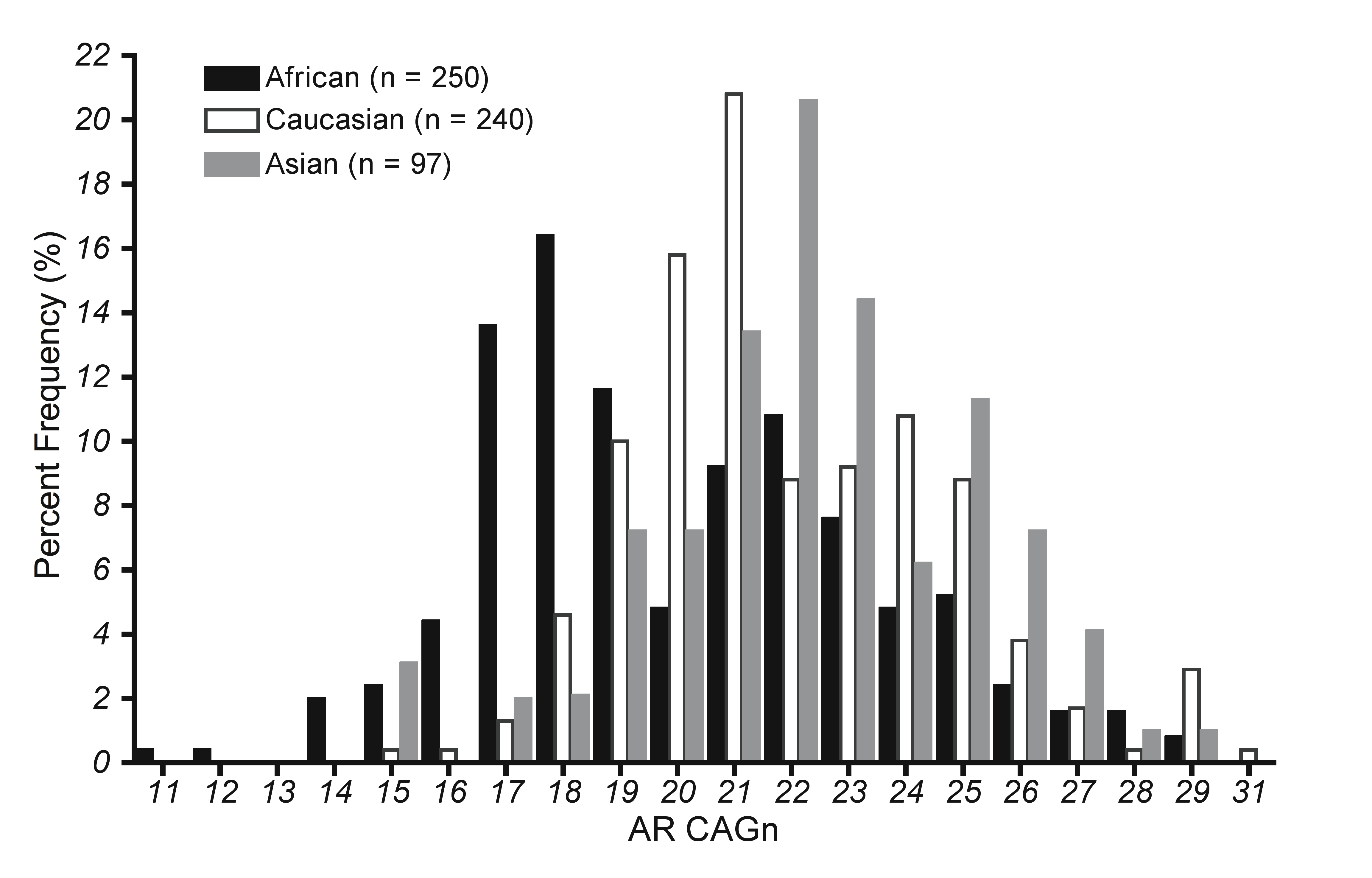
**

Androgen receptor polyglutamine repeat length (AR CAGn) percent allele frequency among three human populations (African, black; Caucasian; white; Asian; grey). Mean repeat length and standard deviation for African, Caucasian, and Asian groups were 20.1 + 0.2, 22 + 0.2, and 22.4 + 0.3, respectively. Number of individuals sampled for each group displayed in the legend. Figure generated based on data from Edwards et al. (1992).
