# Supplementary material for: Androgen receptor polyglutamine repeat number: models of selection and disease susceptibility
Source: Evol Appl. 2012 Jun 11;6(2):180–96. doi: 10.1111/j.1752-4571.2012.00275.x (PMC3586616; doi:10.1111/j.1752-4571.2012.00275.x)
Supplement: Supplementary file 2 [file eva0006-0180-SD2.docx]

| **Phenotype (by Disease Class)** | **Database or Source** | | | | **Androgens and human health…** |
| --- | --- | --- | --- | --- | --- |
|  |  | | | |  |
|  | ARDB | GAD | OMIM | RST |  |
| **Cancer** |  |  |  |  | **Androgens and Human Cancer…** |
| Breast | +/- | +/-/ns | + | +/-/ns | - Hormones, including androgens, often drive cell growth and proliferation, and are important in the etiology of tumorigenesis in a number of human tissues, including in the prostate, breast, endometrium, ovary, testis, and thyroid gland (Jenster 1999; Henderson and Feigelson 2000; Hakimi et al. 1996). - Sequence changes in the androgen receptor gene (including those in polyglutamine repeat number) are frequently associated with cancer progression and may be under positive selection in cancer cell lineages (Ferro et al. 2000). - Even when not directly involved in tumorigenesis, androgens can indirectly drive cell proliferation via aromatization to estrogens (e.g. breast cancer; Maggiolini et al. 1999). - The nature of the association between androgens and cancer is complex. There is evidence for protective or sex-dependant effects of androgens in some cancers (Ferro et al. 2002). |
| Colorectal | - | + |  | + |  |
| Endometrial | + | +/ns |  | +/-/ns |  |
| Esophageal | - | ns |  | ns |  |
| Head and Neck | + | + |  | + |  |
| Hepatocellular |  | - |  | + |  |
| Ovarian | - | -/ns |  | +/-/ns |  |
| Prostate | - | +/-/ns | -/ns | -/ns |  |
| Testicular |  |  |  | ns |  |
|  |  |  |  |  |  |
| **Sexual Development and Identity** |  |  |  |  | **Androgens and Sexual Development and Identity…** |
| Androgen Insensitivity Syndrome (Partial or Complete) |  | + | + |  | - During early fetal development, androgens produced by the testis are essential in the organizational and ontogenic processes involved in sexual differentiation and male-sexual development (Arnold and Breedlove 1985; Swerdloff et al. 1992). - Fetal and pre-pubertal androgen levels direct masculinization and genital development (Chang et al., 2002), and are believed to affect gender identity and sex-specific behaviours prior to and following puberty (Berenbaum et al., 2011; Pardridge et al. 1982; Veale et al., 2010). - While testosterone appears to affect sexual interest and responsiveness (i.e. libido) in both sexes, the relationship between androgens and sexual drive in females may be more complex (Jang and Davis 2002; Bancroft 2002; Bancroft 2003). |
| Genital Abnormalities |  |  | + | +/-/ns |  |
| Homosexuality |  |  | ns |  |  |
| Klinefelter’s Syndrome (phenotype) |  |  | + | + |  |
| Precocious Pubarche or Adrenarch |  |  | - | - |  |
| Response to Potential Mates (Males) |  | - |  |  |  |
| Transexualism |  | +/ns |  |  |  |
| Undermasculinization |  |  | + |  |  |
| **Reproductive Functioning** |  |  |  |  | **Androgens and Reproductive Functioning…** |
| Benign Prostatic Hyperplasia |  | +/-/ns | - |  | - Following puberty, androgens continue to have activational effects on virility and reproductive health and behaviour in both males and females (Bancroft 2002). - Male sperm production, maturation, and fertility are under androgenic control (Collins and Chang 2002). - In females, androgen excess is the most commonly diagnosed endocrinological disorder, and ovarian hyperandrogenism associated with polycystic ovary syndrome (PCOS), which affects 5% of women in the developed world, is associated with infertility, anovulation and ovarian and endometrial cancers (Legro 2003; Knochenhauer et al. 1998). |
| Endometriosis | - | - |  |  |  |
| Infertility | +/- | +/ns | + | +/-/ns |  |
| Azoospermia |  | +/ns | + | +/ns |  |
| Oligospermia |  | +/-/ns | + | +/-/ns |  |
| Ovarian Hyperandrogenism |  | - | - |  |  |
| Polycystic Ovarian Syndrome |  | -/ns | -/+ | -/ns |  |
| Pre-eclampsia |  |  |  | -/ns |  |
| Premature Ovarian Failure |  | + |  |  |  |
| Sperm X:Y (response to POPs) |  | - |  |  |  |
| Uterine Leiomyomas |  | - |  |  |  |
| Urolithiasis |  | + |  |  |  |
| **Neurology, Cognition and Behaviour** |  |  |  |  | **Androgens and Neurology, Cognition, and Behaviour…** |
| ADHD, Conduct Disorder |  | - |  |  | - The ontogenic effects of androgens extend to include sexually dimorphic brain development, differences in cognitive functioning, and behaviour (Swerdloff et al. 1992). - Androgen receptors are highly expressed in a number of regions in the brain and central nervous system, and there is evidence for a role of androgens in visual and verbal memory (Cherrier and Craft 2003), cognitive functioning (Kovacs et al. 2009), social behaviour (Eisenegger et al., 2011), and neuroprotection (Hammond et al. 2001). - The observed effects of androgens may be direct via testosterone or its metabolites or via the aromatization of testosterone to estrodiol in the in the brain or elsewhere (Cherrier and Craft 2003). - Androgens, in the form of endogenous testosterone levels have also been associated with dominant, aggressive, risky and anti-social behaviour in men (Mazur and Booth 1998; Eisenegger et al., 2011). - Females with borderline personality disorder were found to have elevated testosterone and a higher occurrence of PCOS than controls (Roepke et al. 2010), and baseline levels of the androgen dehydroepiandrosterone (DHEA), were found to be significantly higher in schizophrenic patients than in controls (Strous et al. 2004). |
| Alzheimer’s Disease | - | + |  | - |  |
| Alcohol Withdrawal |  | - |  |  |  |
| Amygdala Reactivity |  | - |  |  |  |
| Autism | - | - |  |  |  |
| Cognitive Performance, Memory |  | -/ns |  | - |  |
| Depression |  | - |  | - |  |
| Extraversion | - | - |  |  |  |
| Lateralization and Handedness |  |  |  | +/- |  |
| Migraines |  | ns |  | ns |  |
| Myelin Growth (Adolescents) |  | - |  |  |  |
| Neuroticism | - | - |  |  |  |
| Psychoticism |  |  |  | -/ns |  |
| SBMA (Kennedy’s Disease) | + | + | + | + |  |
| Schizophrenia |  |  |  | ns |  |
| Violent Behaviour | - | - |  | - |  |
| **Metabolism and Cardiovascular Function** |  |  |  |  | **Androgens and Metabolism and Cardiovascular Function…** |
| Coronary Disease |  | - | - | -/ns | - Serum testosterone levels are inversely correlated to factors associated with obesity, insulin resistance, and cardiovascular disease, including body mass index and visceral fat deposit patterns (Mårin and Arver 1998), and serum leptin and insulin levels (Büchter et al. 1999). - Hypogonadal men exhibit higher body fat content than eugonadal controls, an effect which decreases with supplemental T, while obese subjects show lower serum T than non-obese controls (Nieschlag et al. 2004) - The actual relationship between androgens and cardiovascular disease risk appears complex, with androgens conferring both protective and adverse effects, effects that may in some cases be sex-dependant (Hanke et al. 2001; Weidemann and Hanke 2002; Wu and von Eckardstein 2003). |
| Hypertrophic Cardiomyopathy |  | -/ns |  |  |  |
| Hypertension | - | +/ns |  |  |  |
| Insulin Resistance |  | +/-/ns |  | + |  |
| Low Density Lipoprotein Levels |  | - |  |  |  |
| Muscle Mass | - | +/ns |  | + |  |
| Metabolic Syndrome, Obesity |  | +/-/ns |  | + |  |
| Type 1 Diabetes |  | ns |  | ns |  |
| Type 2 Diabetes |  | ns |  |  |  |
| Platelet Reactivity | + |  |  |  |  |
| **Aging, Immunity and Endocrine Function** |  |  |  |  | **Androgens and Aging, Immunity, and Endocrine Function…** |
| Acne | - | - | - | - | - The decline in testosterone in aging males, and corresponding decreases in bone mineral density and muscle mass (Dillon et al. 2010), are well documented (reviewed by Morley 2001). Aging in females is also associated with declines in testosterone, and similar effects on bone and muscle mass, and libido (Morley and Perry 2003). - Humoral immune function, host defense, and autoimmunity are all sexually dimorphic, with females and castrated males demonstrating greater resistance to sepsis and defense against parasites, and females showing far higher rates of autoimmune diseases, including rheumatoid arthritis and lupus (Beery 2003; Olsen and Kovacs 2002; Marriott and Huet-Hudson 2006). - Sebaceous glands and hair follicles are androgen target tissues, and acne, hirusitism, and androgenic alopecia are androgen-dependant dermatological conditions (Uno et al. 2002). - Owing to positive and negative physiological feedback loops and the interactive nature of endocrine systems, androgen levels affect (and are affected by) the levels and activity of other hormones both by direct and indirect (e.g. via affecting sex hormone-binding globulin levels) mechanisms (Norman and Litwack 1997). |
| Androgen Levels |  | +/-/ns | -/ns | - |  |
| Alopecia | - | -/+/ns |  | - |  |
| Aging Male Symptoms |  | + |  |  |  |
| Estrogen Levels |  | +/ns |  |  |  |
| Hirusitism | - |  | -/ns | - |  |
| Leptin Levels |  | +/- |  | + |  |
| Low Bone Mass, Osteoporosis | +/- | +/- | + | +/-/ns |  |
| Osteoarthritis |  | - | + | ? |  |
| Rheumatoid Arthritis | - | ns |  | - |  |
|  |  | | | |  |

**Appendix B: Phenotypes and diseases of androgens and the AR CAGn**

List of phenotypes (by disease type or class) which have been investigated in relation to the exon 1 polyglutamine repeat length in the androgen receptor (AR CAGn), according to three databases and one comprehensive review (ARDB, Androgen Receptor Mutations Database; GAD, Genetic Association Database; OMIM, Online Inheritance In Man; RST, Rajender et al. 2007). Results describe the breadth and range of diseases investigated when the databases were accessed, and were used as a starting point for literature collection. Plus (+), minus (-) signs indicate the direction of the association (or lack of association, ns) identified by accompanying studies (which may be a single study or may represent multiple studies). Studies cited by databases may overlap, resulting in the same study (or studies) being cited by more than one database source. Phenotype or disease names are based on those used by the source, and thus a study examining multiple phenotypes may be referred to more than once. ADHD = Attention-deficit hyperactivity disorder; POPs = Persistant Organic Pollutants; PCOS = Polycystic Ovary Syndrome; SBMA = Spinal and bulbar muscular atrophy.

References

Arnold, Arthur P., and S. Marc Breedlove. 1985. “Organizational and activational effects of sex steroids on brain and behavior: A reanalysis.” *Hormones and Behavior* **19**(4): 469-498.

Bancroft, J. 2003. Androgens and sexual function in men and women. In *Androgens in health and disease*, ed. C.J. Bagatell and William J. Bremner, 259-290. Totowa, NJ: Humana Press.

Bancroft, John. 2002. “Biological factors in human sexuality.” *Journal of Sex Research* **39**(1): 15-21.

Beery, Theresa A. 2003. “Sex differences in infection and sepsis.” *Critical Care Nursing Clinics of North America* **15**(1): 55-62.

Berenbaum, Sheri A., Judith E. Owen Blakemore, and Adriene M. Beltz. 2011. “A Role for Biology in Gender-Related Behavior.” *Sex Roles* **64** (11-12): 804-825.

Büchter, D, H M Behre, S Kliesch, A Chirazi, E Nieschlag, G Assmann, and A von Eckardstein. 1999. “Effects of testosterone suppression in young men by the gonadotropin releasing hormone antagonist cetrorelix on plasma lipids, lipolytic enzymes, lipid transfer proteins, insulin, and leptin.” *Experimental and Clinical Endocrinology & Diabetes: Official Journal, German Society of Endocrinology [and] German Diabetes Association* **107** (8): 522-529.

Chang, J.A., H.T. Nguyen, and T.F. Lue. 2002. Androgens in penile development, penile erection, and erectile dysfunction. In *Androgens and androgen receptor: mechanisms, functions, and clinical applications*, ed. Chawnshang Chang, 289-298. Norwell, MA: Springer.

Cherrier, M.M., and S.C. Craft. 2003. Androgens and Cognition. In *Androgens in health and disease*, ed. C.J. Bagatell and William J. Bremner, 291-309. Humana Press.

Collins, L.L., and Chawnshang Chang. 2002. Androgens and the androgen receptor in male sex development and fertility. In *Androgens and androgen receptor: mechanisms, functions, and clinical applications*, ed. Chawnshang Chang, 299-323. Norwell, MA: Springer.

Dillon, E. Lichar, William J. Durham, Randall J. Urban, and Melinda Sheffield-Moore. 2010. “Hormone treatment and muscle anabolism during aging: Androgens.” *Clinical Nutrition* **29**(6): 697-700.

Eisenegger, Christoph, Johannes Haushofer, and Ernst Fehr. 2011. “The role of testosterone in social interaction.” *Trends in Cognitive Sciences* **15**(6): 263-271.

Ferro, Paola, Maria G Catalano, Mariangela Raineri, Gigliola Reato, Raffaella dell’ Eva, Mauro Risio, Robin Foà, Nicoletta Fortunati, and Ulrich Pfeffer. 2000. “Somatic alterations of the androgen receptor CAG repeat in human colon cancer delineate a novel mutation pathway independent of microsatellite instability.” *Cancer Genetics and Cytogenetics* **123**(1): 35-40.

Ferro, Paola, Maria G. Catalano, Raffaella Dell’Eva, Nicoletta Fortunati, and Ulrich Pfeffer. 2002. “The androgen receptor CAG repeat: a modifier of carcinogenesis?” *Molecular and Cellular Endocrinology* **193**(1-2): 109-120.

Hakimi, J.M., R.H. Rondinelli, M.P. Schoenberg, and E.R. Barrack. 1996. Androgen receptors in human prostate cancer: heterogeneous expression, gene mutations, and polymorphic variants. In *Hormones and cancer*, ed. Wayne V. Vedeckis, 445-492. Boston, MA: Birkhäuser..

Hammond, Jennifer, Quynh Le, Cynthia Goodyer, Morrie Gelfand, Mark Trifiro, and Andrea LeBlanc. 2001. “Testosterone-mediated neuroprotection through the androgen receptor in human primary neurons.” *Journal of Neurochemistry* **77**(5): 1319-1326.

Hanke, Hartmut, Christina Lenz, Beate Hess, Klaus-Dieter Spindler, and Wolfgang Weidemann. 2001. “Effect of Testosterone on Plaque Development and Androgen Receptor Expression in the Arterial Vessel Wall.” *Circulation* **103**(10): 1382-1385.

Henderson, Brian E., and Heather Spencer Feigelson. 2000. “Hormonal carcinogenesis.” *Carcinogenesis* **21**(3): 427 -433.

Jang, C., and S.R. Davis. 2002. Androgen deficiency and abnormality in women. In *Androgens and androgen receptor: mechanisms, functions, and clinical applications*, ed. Chawnshang Chang, 477-488. Norwell, MA: Springer.

Jenster, G. 1999. “The role of the androgen receptor in the development and progression of prostate cancer.” *Seminars in Oncology* **26**(4): 407-421.

Knochenhauer, E. S., T. J. Key, M. Kahsar-Miller, W. Waggoner, L. R. Boots, and R. Azziz. 1998. “Prevalence of the Polycystic Ovary Syndrome in Unselected Black and White Women of the Southeastern United States: A Prospective Study.” *Journal of Clinical Endocrinology & Metabolism* **83**(9): 3078 -3082.

Kovacs, Denes, Evangelos Vassos, Xiehe Liu, Xueli Sun, Junmei Hu, Gerome Breen, Peter Tompa, David A. Collier, and Tao Li. 2009. “The androgen receptor gene polyglycine repeat polymorphism is associated with memory performance in healthy Chinese individuals.” *Psychoneuroendocrinology* **34**(6): 947-952.

Legro, R.S. 2003. Androgen excess disorders in women. In *Androgens in health and disease*, ed. C.J. Bagatell and William J. Bremner, 123-139. Humana Press.

Maggiolini, Marcello, Olivier Donzé, Elisabeth Jeannin, Sebastiano Andò, and Didier Picard. 1999. “Adrenal Androgens Stimulate the Proliferation of Breast Cancer Cells as Direct Activators of Estrogen Receptor α.” *Cancer Research* **59**(19): 4864 -4869.

Mårin, P, and S Arver. 1998. “Androgens and abdominal obesity.” *Baillière’s Clinical Endocrinology and Metabolism* **12**(3): 441-451.

Marriott, Ian, and Yvette M. Huet-Hudson. 2006. “Sexual Dimorphism in Innate Immune Responses to Infectious Organisms.” *Immunologic Research* 34 (3): 177-192. doi:10.1385/IR:34:3:177.

Mazur, Allan, and Alan Booth. 1998. “Testosterone and Dominance in Men.” *Behavioral and Brain Sciences* **21**(03): 353-363.

Morley, John E. 2001. “Androgens and aging.” *Maturitas* **38**(1): 61-71.

Morley, John E., and H. Mitchell Perry. 2003. “Androgens and Women at the Menopause and Beyond.” *The Journals of Gerontology Series A: Biological Sciences and Medical Sciences* **58**(5): M409 -M416.

Norman, Anthony W., and Gerald Litwack. 1997. *Hormones*. Academic Press.

Olsen, N.J., and W.J. Kovacs. 2002. Androgens in immunology and autoimmune diseases. In *Androgens and androgen receptor: mechanisms, functions, and clinical applications*, ed. Chawnshang Chang, 279-288. Norwell, MA: Springer, October 31.

Pardridge, William M., Roger A. Gorski, Barbara M. Lippe, and Richard Green. 1982. “Androgens and Sexual Behavior.” *Annals of Internal Medicine* **96**(4): 488 -501.

Roepke, Stefan, Andreas Ziegenhorn, Julia Kronsbein, Angela Merkl, Scharif Bahri, Julia Lange, Horst Lübbert, Ulrich Schweiger, Isabella Heuser, and Claas-H. Lammers. 2010. “Incidence of polycystic ovaries and androgen serum levels in women with borderline personality disorder.” *Journal of Psychiatric Research* **44**(13): 847-852.

Strous, Rael D., Rachel Maayan, Raya Lapidus, Leonid Goredetsky, Ella Zeldich, Moshe Kotler, and Abraham Weizman. 2004. “Increased circulatory dehydroepiandrosterone and dehydroepiandrosterone-sulphate in first-episode schizophrenia: relationship to gender, aggression and symptomatology.” *Schizophrenia Research* **71** (2-3): 427-434.

Swerdloff, Ronald S., Christina Wang, Melissa Hines, and Roger Gorski. 1992. “Effect of androgens on the brain and other organs during development and aging.” *Psychoneuroendocrinology* **17**(4): 375-383.

Uno, H., S. Itami, S. Inui, H. Pan, E. Chang, S. Takayasu, F. Ye, et al. 2002. Androgens and androgen receptor in dermatology. In *Androgens and androgen receptor: mechanisms, functions, and clinical applications*, ed. Chawnshang Chang, 411-476. Springer.

Veale, Jaimie F., David E. Clarke, and Terri C. Lomax. 2010. “Biological and psychosocial correlates of adult gender-variant identities: A review.” *Personality and Individual Differences* **48**(4): 357-366.

Weidemann, Wolfgang, and Hartmut Hanke. 2002. “Cardiovascular Effects of Androgens.” *Cardiovascular Drug Reviews* **20**(3): 175-198.

Wu, F.C.W., and A. von Eckardstein. 2003. Androgens and coronary artery disease. In *Androgens in health and disease*, ed. C.J. Bagatell and William J. Bremner, 191-220. Totowa, NJ: Humana Press.
